# Mucositis, peri-implantitis, and survival and success rates of oxide-coated implants in patients treated for periodontitis 3- to 6-year results of a case-series study

**DOI:** 10.1186/s40729-017-0110-6

**Published:** 2017-11-28

**Authors:** Reiner Mengel, Theresa Heim, Miriam Thöne-Mühling

**Affiliations:** 1 Department of Prosthetic Dentistry, School of Dental Medicine, Philipps-University, Marburg/Lahn, Germany; 2 Gruben, Brandenburg Germany

**Keywords:** Dental implants, Mucositis, Peri-implantitis, Periodontitis, Survival rate

## Abstract

**Aim:**

The aim of this case-series study is to evaluate the prevalence of mucositis, peri-implantitis, and survival and success rates of oxide-coated implants in subjects treated for periodontitis.

**Materials and methods:**

Twenty-four subjects treated for generalized chronic periodontitis (GCP) and five treated for generalized aggressive periodontitis (GAP) were orally rehabilitated with a total of 130 dental implants. Subjects were examined 2 to 4 weeks prior to extraction of non-retainable teeth and at insertion of superstructure. Additional examinations were performed during a 3-month recall schedule over a 3- to 6-year follow-up period. Radiographs were taken after insertion of the superstructure and 1, 3, and 5 years later.

**Results:**

The results showed implant survival rates of 97.1% in GCP subjects versus 96.2% in GAP subjects. The implant success rate was 77.9% in GCP subjects and 38.5% in GAP subjects. In GCP subjects, mucositis was present in 7.7% and peri-implantitis in 12.5% of the implants. In GAP subjects, 28.0% of the implants showed mucositis and 32.0% peri-implantitis. Implant failure, mucositis, and peri-implantitis were more evident in GAP subjects. Peri-implantitis was more prevalent for implants in the maxilla and implants >10 mm. After 5 years, the mean peri-implant bone loss in GAP subjects was 2.89 mm and in GCP subjects 1.38 mm.

**Conclusions:**

Periodontally diseased subjects treated in a supportive periodontal therapy can be successfully rehabilitated with oxide-coated dental implants for a follow-up period of 3- to 6-years. Implants in the maxilla and GAP subjects were more susceptible to mucositis and peri-implantitis, with lower implant survival and success rates.

## Background

In recent years, a great number of different implant systems varying in materials, surface structure, and macroscopic design have been introduced to the dental market [1]. In studies using implants with modified surfaces, it was concluded that rough surfaces induce a stronger initial bone response, achieve stability more rapidly, and integrate more fully with extant bone [2–6]. Dental implants with oxide-coated (anodised) surfaces have demonstrated, in histologic and histomorphometric examinations, that the newly formed bone infiltrates the pores of the surface oxide layer and thereby establishes a strong interlock between the bone and oxidized implant [7–9]. The oxide-coated implant surface is categorized as “moderately rough,” typically with a thickened titanium oxide layer of high crystallinity and phosphorous content.

A prospective long-term clinical study on implants with oxide-coated surfaces revealed an implant survival rate of 99.2% and mean marginal bone loss of 0.7 ± 1.35 mm after 10 years of function [10]. Only 1.9% of the implants showed significant marginal bone loss (> 3 mm) together with bleeding on probing and suppuration. In a retrospective study, no difference could be found when comparing the clinical performance (survival rate, marginal bone loss, presence of bleeding, and probing depth) of turned versus oxide-coated surface implants after 5 years of loading [11]. In a 9-year study with an immediate loading protocol, implants with oxide-coated surfaces achieved a 10% higher survival rate compared to turned surface implants [12].

These results indicate that the survival rate of dental implants in long-term studies seems to be high, irrespective of surface type. However, the influence of the implant surface type on the development and progression of mucositis and peri-implantitis especially in periodontally diseased subjects remains largely unknown. In animal studies, it has been suggested that oxide-coated implants are more susceptible to mucositis and peri-implantitis [13–15]. Whether or to what extent these findings might be translated to humans is yet unknown. A Cochrane review found no evidence of a superior long-term success that could be attributed to any one type of implant surface [1]. Furthermore, the review concluded that there are limited data suggesting that implants with relatively smooth surfaces are less susceptible to peri-implantitis-induced bone loss.

The aim of this long-term clinical study on partially edentulous subjects treated for periodontal disease was to evaluate the prevalence of mucositis and peri-implantitis and to determine the survival and success rates of dental implants with oxide-coated surfaces.

## Materials and methods

### Study population

A total of 29 partially edentulous subjects were consecutively recruited from the Dental School of Medicine, Philipps-University, Marburg, Germany between April 2010 and April 2013 (Table 1). Subjects were excluded for the following reasons: history of systemic disease (e.g., cardiovascular diseases, diabetes mellitus, osteoporosis), pregnancy, untreated caries, current orthodontic treatment, continuous drug administration, and psychiatric disorders. Systemic diseases were assessed by an internist.

**Table 1:** Implants in study population

	GCP	GAP
Patient	24	5
Sex		
Female	15	3
Male	9	2
Age		
< 50 years	2	2
> 50 years	22	3
Implant system		
Nobelspeedy Replace RP	49	17
Nobelspeedy Replace NP	16	4
Nobel Replace Straight Groovy	29	5
Nobelspeedy Groovy	10	0
Topography		
Anterior maxilla	26	12
Posterior maxilla	36	10
Anterior mandible	18	0
Posterior mandible	24	4
Superstructure		
Single crowns	52	4
Removable	41	21
Fixed bridges	11	0
Bone quality		
1	3	–
2	97	21
3	4	5
Degree of atrophy		
A	41	12
B	28	14
C	35	–

All subjects were treated for periodontitis at the Dental School of Medicine, Philipps-University, Marburg, Germany. Periodontal treatment was followed by a 3-month recall schedule for 3 to 6 years. Each recall session comprised oral hygiene control with motivation and instruction, subgingival scaling, and root planing at tooth surfaces with probing depth (PDs) > 4 mm, and bleeding on probing (BOP). Preceding implant placement, non-retainable teeth were removed and subgingival scaling and root planing were performed for residual teeth where necessary. Six months after tooth removal, the residual teeth showed healthy periodontal tissue with PDs ≤ 3 mm and no BOP.

Periodontal disease was diagnosed according to the criteria of the American Academy of Periodontology [16]. The clinical and radiological findings in the recall schedule before insertion of the implants were the basis to distinguish between generalized chronic periodontitis (GCP) and generalized aggressive periodontitis (GAP). Twenty-four subjects (9 males and 15 females; mean age, 63 years) with GCP displaying more than 30% of sites affected, with bone loss < 0.2 mm per year. Five subjects (two males and three females; mean age, 31 years) with GAP displaying more than 30% of sites affected, with bone loss > 0.2 mm per year.

### Implant placement and prosthesis

At total, 130 implants with oxide-coated surfaces (Nobel Replace Straight Groovy; Nobel Speedy Groovy; Nobel Speedy Replace, Nobel Biocare, Zürich, Switzerland) were placed with a length of 10 to 15 mm and a diameter of 3.5 or 4 mm. In GCP subjects 104 implants were inserted, and in GAP subjects, 26 implants (Table 1).

In both groups, the bone quality and atrophy of the alveolar bone were classified during implant insertion according to Lekholm and Zarb [17].

Second-stage surgery was performed in the maxilla after 6 months and in the mandible after 3 months. Implant placement and second-stage surgery were performed by a single periodontist (R.M.).

About 4 weeks after the final abutments were placed, GCP subjects were rehabilitated with single crowns, implant-supported bridges, or removable superstructures, according to the Marburg double crown system [18] (Table 1). In GAP subjects, single crowns or removable superstructures (Marburg double crown system) were inserted. All prosthetic appliances were provided at the Dental School of Medicine, Philipps-University, Marburg, Germany. All crowns and bridges were cemented and solely porcelain-fused-to-metal restorations.

### Clinical parameters

At each session, the Gingival Index (GI) [19], Plaque Index (PI) [20], PDs, BOP, gingival recession (GR), and clinical attachment level (CAL) were evaluated at four sites (mesial, distal, buccal, and lingual/palatinal) on the teeth and implants. The CAL was measured at the teeth from the cement-enamel junction to the base of the pocket. For implants, the upper edge of the corresponding final abutment served as the top reference point. Trauma to peri-implant tissue was avoided by waiting 1 year after implant placement before measuring probing depths.

The clinical examinations were performed by four examiners (all dentists, formally affiliated with the Dental School of Medicine, Philipps-University, Marburg, Germany) before study initiation, each examiner was calibrated for intra- and interexaminer reproducibility using duplicate measurements of a minimum of 50 sites in at least five subjects. The correlation coefficients were 0.90 to 0.99 for intraexaminer reproducibility and 0.91 to 0.95 for interexaminer reproducibility.

### Radiographic examination

Standardized radiographs of the teeth and implants were taken by two persons using the parallel technique [21]. These radiographs were obtained immediately after insertion of the superstructure (baseline for mucositis and peri-implantitis evaluation) and at 1, 3, and 5 years thereafter. The digitized radiographs were evaluated using a computer software (Planmeca Romexis Version 3.0.1, Planmeca, Helsinki, Finland). Bone loss was determined in relative terms at the mesial and distal tooth surfaces by measuring the distance from the CEJ to the apex. The distance from the marginal bone level to the upper edge of the implant was measured (in mm) at the mesial and distal implant surfaces and related to the implant thread. All radiographs were analyzed by an independent masked examiner.

### Study follow-up schedule

All patients received a supportive periodontal therapy at the Dental School of Medicine, Philipps-University, Marburg, in the course of the observation period. The first clinical examination was 2 to 4 weeks before the non-retainable teeth were extracted. The periodontally healthy residual dentition and the implants were evaluated immediately after the superstructure was inserted. Subsequently, the subjects were followed up at 3-month intervals for 3 to 6 years. At each follow-up session, the clinical parameters were recorded and subjects were remotivated and reinstructed in effective oral hygiene. In addition, the teeth and implants were cleaned professionally. Supragingival deposits were removed, followed by polishing with rubber cups and polishing paste. Subgingival debridement was performed in the teeth and implants with PDs > 4 mm and BOP positive. In the teeth, conventional stainless-steel curettes and ultrasonic devices were used, whereas in implants, plastic curettes and polyether ether ketone-tips for the ultrasonic device were applied to avoid damage of the implant surface.

A functional analysis and medical history were performed at the beginning of the study and reviewed annually.

Cigarette smoking status was self-reported. Subjects were considered smokers if they had been smoking 10 or more cigarettes a day during the past 5 years [22].

### Statistical evaluations

Data analysis was performed with a computerized statistics package (SPSS 12.0.1 for Windows, SPSS). The examined patients were not included in any other publications.

Mean values for clinical and radiologic parameters were determined separately for the implants and the teeth, for both patient groups, and for every visit. Four visits were consolidated for analysis.

The probability of implant loss (implant survival) at a certain time was computed with reference to previously established criteria using a Kaplan-Meier survival curve.

The assessment of implant success, mucositis, and peri-implantitis was performed at the time of radiographic examination 1 year after insertion of the superstructure and 3 and 5 years thereafter.

The implant success rate was defined by the following parameters: no implant movement, no discomfort (pain, foreign body sensation etc.), PDs ≤ 5 mm without BOP, no continuous radiologic translucency surrounding implants, and annual peri-implant bone loss ≤ 0.2 mm 1 year after insertion of the superstructure [23]. Implants that did not meet at least one criterion were considered a failure.

Peri-implant mucositis was defined as PDs ≥ 5 mm with BOP and no bone loss after the first year of loading. Peri-implantitis was defined as PDs > 5 mm with or without BOP and an annual bone loss of > 0.2 mm after the first year of loading.

All technical and surgical complications (e.g., fracture of the abutment screw or superstructure, compromised wound healing) were recorded.

The potential risk factors of gender, implant topography, implant length, type of superstructure, and bone quality and atrophy were analyzed for their correlation with the prevalence of mucositis, peri-implantitis, implant success, and survival. At first, the effect of each risk factor was tested with a univariate regression analysis. A multivariate analysis was performed for risk factors with *P* values of ≤ 0.05 in the univariate analysis. The extent of the effect of a risk factor was indicated with an odds ratio (OR), with a confidence interval (CI) of 95%.

## Results

All 29 subjects were examined over the period of 3 to 6 years (Table 2). For the duration of the observation period, all the remaining teeth were periodontally healthy, with PDs ≤ 3 mm and negative BOP. All subjects were non-smokers, had excellent oral hygiene, attended the follow-up examinations on a regular basis, and had no systemic disease.

**Table 2:** Observation period (patient and implant related)

Years	Patients (total)	GCP patients	GAP patients	Implants (total)	Implants maxilla			Implants mandible		
					Total	GCP	GAP	Total	GCP	GAP
3	29	24	5	130	84	62	22	46	42	4
4	22	17	5	92	55	34	21	37	33	4
5	20	15	5	84	53	32	21	31	27	4
6	17	12	5	74	50	28	21	24	20	4

### Implant survival

In total, four implants (3.1%) were lost during the observation period. In a GAP subject (male), one implant (left upper first bicuspid) was removed during second-stage surgery because of mobility. In two GCP subjects (one male and one female), two implants with single crowns (right upper first bicuspid and left lower first molar) were removed after 53 and 68 months due to peri-implantitis. One implant with a single crown (right lower first molar) of a GCP subject (female) fractured 27 months after loading. The implant survival rate was 96.2% in GAP subjects and 97.1% in GCP subjects.

### Mucositis

Nine subjects (31.0%) showed mucositis in 15 implants (11.6%) (Table 3). Three GAP subjects displayed mucositis in seven implants (28.0%), compared to eight implants (7.7%) in six GCP subjects. In more than 70% of the implants, a mucositis was first diagnosed after 3 years of loading.

**Table 3:** Risk factors for mucositis

	Mucositis	*n*	Univariate analyses			Multivariate analyses		
			OR	(95% CI)	*P*	OR	(95% CI)	*P*
Subjects								
GAP	3	5	4.500	(0.601; 3.708)	0.138			n.s.
GCP	6	24						
Female	7	18	2.864	(0.473; 17.351)	0.231			n.s.
Male	2	11						
Implants								
GAP	7	25	4.667	(1.504; 4.482)	0.010	4.672	(1.447; 15.080)	0.012
GCP	8	104						
Female	13	76	5.269	(1.135; 4.393)	0.013	5.267	(1.104; 25.122)	0.016
Male	2	53						
Superstructure								
Single crown	6	56	0.897	(0.509; 1.583)	0.708			n.s.
Fixed bridge	1	11						
Removable	8	62						
Bone quality								
1	0	3	2.769	(0.506; 15.168)	0.203			n.s.
2	13	118						
3	2	8						
Degree of atrophy								
A	8	53	1.609	(0.785; 3.298)	0.179			n.s.
B	5	41						
C	2	35						
Topography								
Ant. maxilla	7	38	2.423	(0.647; 9.074)	0.161			n.s.
Post. maxilla	5	45						
Ant. mandible	0	18						
Post. mandible	3	28						
Implant length								
≥ 10 mm	9	81	0.875	(0.291; 2.631)	0.813			n.s.
< 10 mm	6	48						

Logistic regression analyses showed whether differences were significant (*p* ≤ 0.05)

*CI* confidence interval, *n.s*. non-significant, *OR* odds ratio, *p* significance

In the multivariate implant-related analyses, the risk of mucositis was higher in GAP subjects (OR = 4.672 with *p* = 0.012) and in females (OR = 5.267 with *p* = 0.016).

The uni- and multivariate patient-related analyses did not show significant differences. All other clinical parameters were found to be non-significant in both the implant- and patient-related analyses.

### Peri-implantitis

Seven subjects (24.1%) with 21 implants (16.3%) showed peri-implantitis (Table 4). Three GAP subjects displayed peri-implantitis in 8 implants (32.0%) compared to 13 implants (12.5%) in four GCP subjects. In about 60% of the implants, a peri-implantitis was first diagnosed after 3 years of loading.

**Table 4:** Risk factors for peri-implantitis

	Peri-implantitis	*n*	Univariate analyses			Multivariate analyses		
			OR	(95% CI)	*P*	OR	(95% CI)	*P*
Subjects								
GAP	3	5	7.500	(0.931; 60.427)	0.054			n.s.
GCP	4	24						
Female	4	18	0.762	(0.135; 4.301)	0.759			n.s.
Male	3	11						
Implants								
GAP	8	25	3.294	(1.186; 9.151)	0.027	2.596	(0.720; 9.352)	0.149
GCP	13	104						
Female	9	76	0.459	(0.178; 1.184)	0.105			n.s.
Male	12	53						
Superstructure								
Single crown	7	56	0.881	(0.538; 1.442)	0.613			n.s.
Fixed bridge	4	11						
Removable	10	62						
Bone quality								
1	0	3	21.200	(3.915; 114.798)	0.000	30.896	(5.178; 0.868)	0.056
2	16	118						
3	6	8						
Degree of atrophy								
A	5	53	0.716	(0.404; 1.269)	0.252			n.s.
B	10	41						
C	6	35						
Topography								
Ant. maxilla	9	38	14.286	(1.849; 110.358)	0.000	15.680	(1.914; 128.455)	0.001
Post. maxilla	11	45						
Ant. mandible	0	18						
Post. mandible	1	28						
Implant length								
≥ 10 mm	9	81	7.048	(1.563; 31.782)	0.002	9.555	(1.900; 48.051)	0.001
< 10 mm	6	48						

Logistic regression analyses showed whether differences were significant (*p* ≤ 0.05)

*CI* confidence interval, *n.s*. non-significant, *OR* odds ratio, *p* significance

In the multivariate implant-related analyses, the risk of peri-implantitis was higher in the maxilla (OR = 15.680 with *p* = 0.001) and implants >10 mm (OR = 9.555 with *p* = 0.001). All other clinical parameters were found to be non-significant.

The univariate analyses showed a significantly higher risk for peri-implantitis in GAP subjects (OR = 3.294 with *p* = 0.027) and at implants with bone quality grade 3 (OR = 21.200 with *p* = 0.000). However, these differences were not significant in multivariate analyses.

Both the uni- and multivariate patient-related analyses were non-significant.

### Implant success

The implant success rate was 77.9% for GCP implants and 38.5% for GAP implants. Twenty-two implants (21.2%) failed in 10 GCP subjects (41.7%), and 16 implants (61.5%) failed in (all) GAP subjects (100.0%).

In the multivariate implant-related analyses, implants placed in the maxilla (OR = 3.241 with *p* = 0.022), and in GAP subjects (OR = 4.218 with *p* = 0.006), had a significantly higher risk of failure.

The multivariate patient-related analyses showed a higher risk of implant failure in GAP subjects (OR = 3.032 with *p* = 0.004) (Table 5).

**Table 5:** Implant failure rates

	Failure rate	*n*	Univariate analyses			Multivariate analyses		
			OR	(95% CI)	*P*	OR	(95% CI)	*P*
Subjects								
GAP	5	5	2.400	(1.495; 3.853)	0.006	3.032		0.004
GCP	10	24					(1.131; 8.123)	
Female	11	18	0.364	(0.077; 1.716)	0.194			n.s.
Male	4	11						
Implants								
GAP	16	26	5.964	(2.378; 14.959)	0.000	4.218	(1.515; 1.746)	0.006
GCP	22	104						
Female	23	76	1.128	(0.522; 2.439)	0.758			n.s.
Male	15	54						
Superstructure								
Single crown	15	56	0.952	(0.854; 1.062)	0.368			n.s.
Fixed bridge	5	11						
Removable	18	62						
Bone quality								
1	0	3	4.172	(3.038; 5.731)	0.000			n.s.
2	30	118						
3	9	9						
Degree of atrophy								
A	14	53	0.912	(0.330; 2.496)	0.156			n.s.
B	17	42						
C	8	35						
Topography								
Ant. maxilla	16	38	5.306	(1.901; 14.810)	0.000	3.241	(1.105; 9.512)	0.022
Post. maxilla	17	46						
Ant. mandible	0	18						
Post. mandible	6	28						
Implant length								
≥ 10 mm	9	82	0.485	(0.211; 1.115)	0.080			n.s.
< 10 mm	6	48						

Logistic regression analyses showed whether differences were significant (*p* ≤ 0.05)

*CI* confidence interval, *n.s*. non-significant, *OR* odds ratio, *p* significance

### Radiological evaluation

The mean mesial and distal marginal bone loss after 5 years was 2.19 mm (SD 1.85) in both patient groups. Peri-implant bone loss in GAP subjects was 2.89 mm (SD 1.90) and that in GCP subjects 1.38 mm (SD 1.05).

### Complications

Mechanical complications were observed in two implants in two GAP subjects and in six implants in five GCP subjects. One abutment screw fractured, as well as the veneers of four ceramic crowns, and three abutments loosened and unscrewed. Surgical complications were not seen.

## Discussion

The present study examines the success rates of oxide-coated implants in subjects with treated periodontal disease. Several long-term clinical studies on periodontally healthy subjects have revealed survival rates of 97.1 to 99.2% for oxide-coated implants [10, 24, 25]. The results of the present study show a comparable implant survival rate (96.2% in GAP and 97.1% in GCP subjects) for subjects with treated periodontal disease.

These findings confirm numerous studies indicating that implants with different surfaces in subjects with generalized chronic periodontitis have a survival rate of over 90% after 5 years [23, 26–31]. In a prospective study with GCP subjects, implants with rough surfaces showed a survival rate of 96.0% after an observation period of 11.6 years [32]. However, the implant survival rate for GAP subjects was 80.0%, after a follow-up period of 8.3 years. The lower survival rate of implants in patients with GAP was also present on implants with turned surfaces. In a prospective 5- to 16-year study, the survival rate was only 83.3% [23].

When reflecting the higher survival rate of implants in GAP subjects in the present study, one has to consider the small number of subjects in this group as well as the short follow up of 6 years. In a systematic review, comparing implant survival rates for GAP subjects in long-term studies, it was shown that survival rates ranged from 97.4 to 100% in studies with a follow-up period of < 5 years, falling to 83.3 to 96.0% for studies with longer observation periods [33].

Although implant survival rates given in different studies are comparable, analyzing mucositis and peri-implantitis prevalence is challenging. In the present study, GCP subjects showed mucositis in 7.7% and peri-implantitis in 12.5% of the implants. The GAP group displayed mucositis in 28.0% and peri-implantitis in 32.0% of the implants. A prospective 10-year study that examined GCP subjects with rough surface implants revealed a slightly higher peri-implantitis rate (28.6%) [31]. In a 3- to 16-year study, GAP subjects with turned surfaces implants displayed a higher mucositis (56.0%) and a comparable peri-implantitis rate (26.0%) [23].

These results from long-term clinical studies indicate that oxide-coated implants achieve equivalent survival rates and prevalence of mucositis and peri-implantitis when compared to implants with other surface characteristics. They support the assumption that the implant surface has little influence on the development of mucositis or peri-implantitis. This was subsequently confirmed in a Cochrane review, where in clinical long-term studies no evidence could be found that any specific type of implant system or surface modification conferred superior long-term success [1].

The findings of the present clinical study also allow us to put the previous results obtained from animal studies into context [13–15]. These studies analyzed the effects of ligature-induced peri-implantitis in implants with different surface characteristics placed in Labrador dogs. The results revealed increased marginal bone loss and more soft tissue destruction surrounding oxide-coated implants as compared to implants with other surfaces. These animal studies were subject to critical review [34], with the authors identifying shortcomings pertaining to the statistical analyses. Due to the small number of animals examined (six dogs), it is not possible to draw any valid conclusion regarding clinical application in human subjects. It is also apparent that the results of such animal studies are not wholly predictive of the human scenario [35].

## Conclusions

The results of the present case series study should be interpreted in a critical light because of the small study population. However, it can be concluded that periodontally diseased subjects treated in a supportive periodontal therapy can be successfully rehabilitated with oxide-coated dental implants for a follow-up period of 3 to 6 years. The results suggest that implants in the maxilla and in subjects treated for generalized aggressive periodontitis were more susceptible to developing mucositis and peri-implantitis, with lower implant survival and success rates.
